# Esearch3D: propagating gene expression in chromatin networks to illuminate active enhancers

**DOI:** 10.1093/nar/gkad229

**Published:** 2023-04-06

**Authors:** Maninder Heer, Luca Giudice, Claudia Mengoni, Rosalba Giugno, Daniel Rico

**Affiliations:** Biosciences Institute, Faculty of Medical Sciences, Newcastle University, Newcastle upon Tyne, UK; Department of Computer Science, University of Verona, Strada le Grazie 15, 37134, Verona, Italy; A.I. Virtanen Institute for Molecular Sciences, University of Eastern Finland, Kuopio, Finland; Department of Computer Science, University of Verona, Strada le Grazie 15, 37134, Verona, Italy; Department of Computer Science, University of Verona, Strada le Grazie 15, 37134, Verona, Italy; Biosciences Institute, Faculty of Medical Sciences, Newcastle University, Newcastle upon Tyne, UK

## Abstract

Most cell type-specific genes are regulated by the interaction of enhancers with their promoters. The identification of enhancers is not trivial as enhancers are diverse in their characteristics and dynamic in their interaction partners. We present Esearch3D, a new method that exploits network theory approaches to identify active enhancers. Our work is based on the fact that enhancers act as a source of regulatory information to increase the rate of transcription of their target genes and that the flow of this information is mediated by the folding of chromatin in the three-dimensional (3D) nuclear space between the enhancer and the target gene promoter. Esearch3D reverse engineers this flow of information to calculate the likelihood of enhancer activity in intergenic regions by propagating the transcription levels of genes across 3D genome networks. Regions predicted to have high enhancer activity are shown to be enriched in annotations indicative of enhancer activity. These include: enhancer-associated histone marks, bidirectional CAGE-seq, STARR-seq, P300, RNA polymerase II and expression quantitative trait loci (eQTLs). Esearch3D leverages the relationship between chromatin architecture and transcription, allowing the prediction of active enhancers and an understanding of the complex underpinnings of regulatory networks. The method is available at: https://github.com/InfOmics/Esearch3D and https://doi.org/10.5281/zenodo.7737123.

## Graphical Abstract

**Graphical Abstract:**
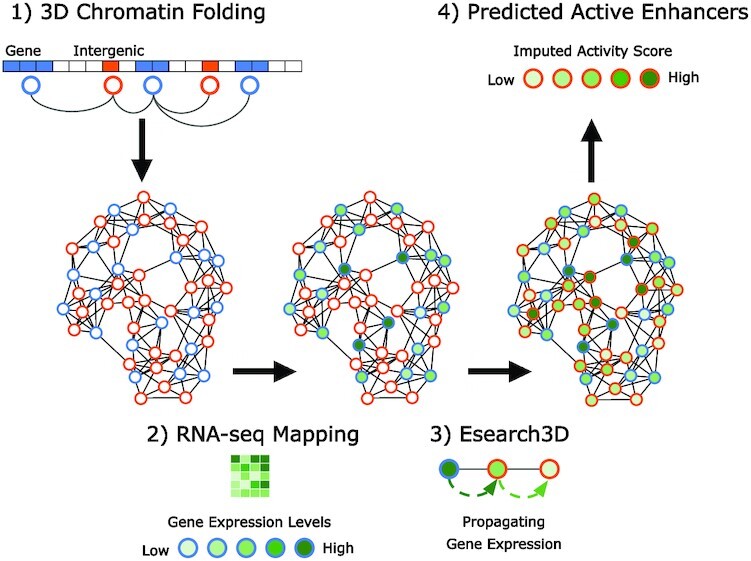
Esearch3D: propagating gene expression in chromatin networks to illuminate active enhancers.

## INTRODUCTION

The non-coding genome contains a diverse collection of regulatory regions including non-coding RNAs, silencers, insulators and *cis/trans*-regulatory elements. Transcriptional enhancers, a class of distal *cis*-regulatory elements, are of particular interest as they are able to direct the cell type-specific transcriptional activity of genes (1). Defining enhancers and disentangling their spatio-temporal localization with promoters is important in the context of understanding a wide variety of biological phenomena such as evolution, homeostasis and disease (2,3). However, current definitions of enhancers are broad and non-specific, while their mode of action is poorly understood. Despite the lower costs and advances in whole-genome sequencing, problems persist in attempts to consolidate the link between enhancers, non-coding genetic variation and protein-coding genes for a great number of diseases. The enhancer characteristics underpinning these difficulties can be broadly summarized by the following points:

- *Enhancers are promiscuous*. A single enhancer can regulate multiple genes (4).
- *Enhancers can be redundant*. Perturbing one enhancer can have a minimal effect on the expression of a gene that may rely on several enhancers (5–7).
- *Enhancer interactions are complex and dynamic*. Enhancers can be located up to, and in some cases beyond, 1 Mb away from their cognate gene (8) and interact with gene promoters in specific three-dimensional (3D) spatio-temporal patterns (9).
- *Enhancer sequence composition is highly heterogeneous*. There is no known conserved enhancer sequence that universally defines all enhancers (2).

To address these difficulties, current methods to find enhancers have sought to identify them through their associated features. One of the most common features are histone modifications. H3K4me1 is associated with enhancers in the active (when co-existing with H3K27ac), inactive and primed (when co-existing with H3K27me3) states (10). Other marks such as H3K4me3 (11), H3K79me2 (12), as well as H3K64ac, H3K122ac and H4K16ac (13), have also been found to be enriched at histones proximal to distinct sets of enhancer sequences. Currently, these marks can be used to identify putative enhancers, but they are correlative and not universally present in all experimentally validated enhancers (13).

Beyond histone marks, coactivators such as CREB-binding protein (CBP) and P300 have been proven to be a prerequisite for the activity of some enhancers by their acetyltransferase activity at H3K27 and recruitment of RNA polymerase II (RNAPII) (14,15). Other methods that are currently used to identify enhancers include enhancer RNAs (eRNAs) that exhibit bidirectional transcription and are quantified by cap analysis of gene expression (CAGE-seq) protocols. FANTOM has catalogued putative enhancers based on this assay (16). In addition to this, self-transcribing active regulatory region sequencing (STARR-seq) has become a popular high-throughput method to experimentally assess the transcriptional-enhancing properties of the DNA sequences in putative enhancers (17–19). Finally, expression quantitative trait loci (eQTLs) can be used to associate non-coding single nucleotide polymorphisms (SNPs) in putative enhancer sequences with changes in gene expression (20,21).

Enhancer-associated features can be used individually or in tandem to identify enhancers, albeit with varying levels of success (22). This highlights the difficulty in both ubiquitously identifying enhancers and assessing the performance of computational models to predict them. A way of improving such predictions is to integrate more data to better inform the model.

The organization of chromatin into higher order hierarchical structures such as A/B compartments, topologically associated domains (TADs) and enhancer–promoter loops has been associated with orchestrating cell type-specific gene regulation by localizing regulatory enhancers to promoters in specific spatio-temporal patterns (3,23). These patterns of connectivity can be uncovered by methods known as chromosome conformation capture (3C) such as Hi-C, capture-Hi-C, ChIA-PET or HiChIP (24). These specific patterns of 3D chromatin conformation add yet another layer of complexity to the genome and represent a feature from which the spatio-temporal localization of enhancers with promoters can be determined. Indeed, there are some pioneering predictive algorithms that have sought to leverage this information to link enhancers with their target genes in specific cell lines. The activity-by-contact (ABC) model uses Hi-C data, gene expression, accessible chromatin information and active histone marks to predict promoter–enhancer interaction (25). Enformer exploits deep learning models trained with massive collections of chromatin and gene expression data to predict gene expression effects of DNA sequence variation at distal regulatory regions using DNA sequence as the sole input (26). The main challenge of these methods is the large amount of training data that is required.

A caveat of using 3C data from bulk cell populations is that it provides a snapshot of the most frequent chromatin interactions and is therefore insensitive to the dynamic and transient nature of many enhancer–promoter interactions. Additionally, single-cell Hi-C approaches are still only capturing a very small proportion of the total interactions (27) and do not allow for the comprehensive detection of promoter–enhancer interactions at adequate resolutions. There is also increasing evidence that enhancer–promoter contacts are not a prerequisite for all enhancer activity. For example, transcriptional bursting has been demonstrated to enable the periodic transcription of genes (28), but the temporal dynamics of promoter–enhancer interactions appear to be uncoupled from those of transcription (29). Moreover, enhancer activity has also been observed with a distinct lack of enhancer–promoter proximity (30). These types of interactions are susceptible to be missed as, typically, 3C data are interrogated in a pairwise manner. To demonstrate this, consider the scenario where the transcription of a gene in fragment A is driven by the enhancer found in fragment C and these are linked by their distinct interactions with fragment B. In traditional 3C data representations, the indirect interactions between fragment A and C are lost. Although microscopy approaches can address this, they are limited by their low throughput. Therefore, a useful way to capture these lost pairwise interactions is to represent 3C data as networks. In a network, an interaction between loci A and C can be inferred through their common interaction with loci B that would otherwise be lost using established pairwise methods.

**Figure 1.**
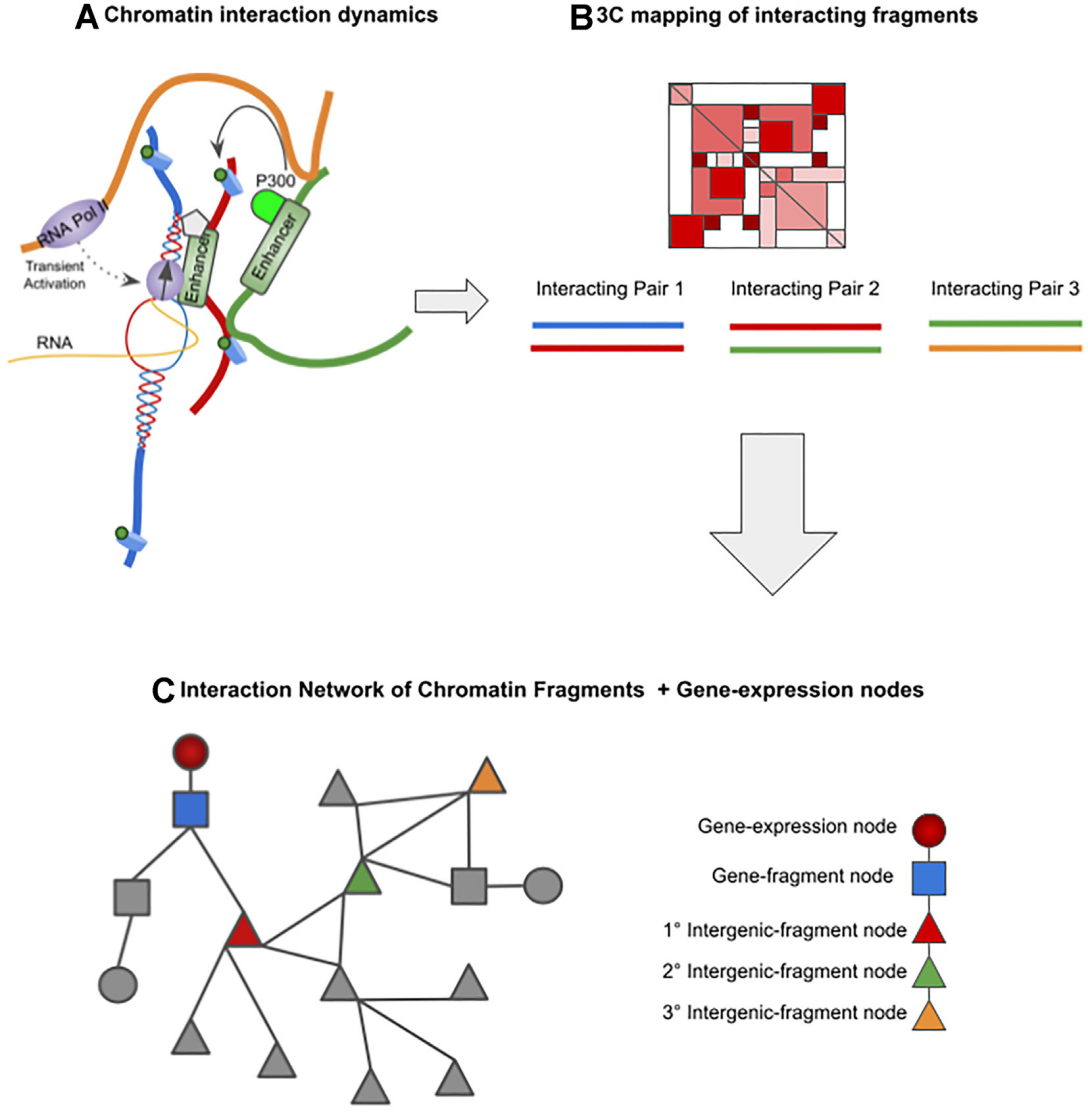
Schematic of converting chromatin structure datasets into networks. (**A**) Enhancers can influence the regulation of promoters directly and indirectly. Here, we show the interactions of four fragments: yellow, blue (containing a transcribed gene), red and green. (**B**) Hi-C and Hi-C-derived methods can capture chromatin interactions but only in a pairwise manner. (**C**) Representation of chromatin fragments as nodes in a network, with their interactions as edges, preserves indirect associations between interacting chromatin fragments and allows global system analyses. Left: a toy CIN is shown, where chromatin fragment nodes are represented as squares when they contain genes (gene-fragment nodes) and as triangles if not (intergenic-fragment nodes). The four coloured fragment nodes correspond to the fragments shown in (A). Esearch3D expands the chromatin network by adding gene-expression nodes (circles) that connect to the fragments containing genes (like the blue fragment). Right: one of the many network paths, showing examples of the three types of nodes.

Representing these 3C data as networks has the potential to capture more chromatin interactions as well as to uncover hidden trends beyond direct interactions such as cooperative regulatory subnetworks (Figure 1). Such advantages of network analysis have already been demonstrated. For example, Sandhu *et al*. constructed a chromatin interaction network (CIN) from RNAPII-mediated interactions describing large-scale organization into chromatin communities with specific function (31). We were able to use CINs and assortativity measures to demonstrate how specific features of the chromatin are enriched in promoter–promoter contacts versus promoter–non-promoter contacts (32). For example, H3K4me3 was more enriched in promoter–promoter interactions, while actively elongating variant RNAPII-S2P was more enriched in promoter–active-enhancer contacts (32). Thibodeau and colleagues have demonstrated that H3K4me3 broad domains and superenhancers in CINs maintained unique and distinct topological properties, including higher connectivity, that could be used to distinguish broad domains from promoters and superenhancers from normal enhancers (33). Finally, recent reports have shown the usefulness of graph-based approaches to detect functional modules in 3C data (34–37).

It is becoming increasingly apparent that the global organization of chromatin and the accumulation of perturbations across the genome can have subtle but tangible effects on gene expression of most expressed genes. An omni-genic model has been proposed, where the activity of almost any gene would affect that of almost every other gene as a result of the small-world properties of regulatory networks (38). This model was proposed as an attempt to explain the multiloci nature of many complex diseases such as Alzheimer's (39) or Crohn's disease (38) that has been uncovered by genome-wide association studies (GWAS). Due to the small-world properties of regulatory networks, most expressed genes in a given cell type would be only a few steps from the nearest core disease gene and thus may have non-zero effects on disease. As these mechanisms probably act to influence the localization of regulatory enhancers to gene promoters, a pan-genomic model based purely on 3D genome interactions has since been proposed to reconcile the strong effects of overexpressing transcription factors in changing cellular phenotypes with the data from eQTL studies (where genetic effects are small but widespread) (40). Focusing solely on the 3D genome network and transcription, this work shows how changing the activity of just one gene can dramatically change the topology of the networks and the overall transcriptional programme This demonstrates clearly that we cannot always study the regulation of a particular gene in isolation (41). Despite this, none of the current methods to identify the enhancers regulating a particular gene takes into account the expression of the other genes being transcribed.

In recent years, network propagation has emerged as a powerful method to integrate biological networks and different types of data, such as the investigation of protein–protein interaction networks to identify new associations between genes and disease or genes and drugs [see review by Cowen *et al.* (42)]. Recent developments of network propagation approaches have shown their usefulness in identifying disease genes from GWAS data (43) and ageing-associated genes (44) or discovering cancer drivers among genes with infrequent cancer mutations (45). Fundamentally, network propagation aims to describe the associations and relationships between the nodes in the network. This type of analysis is not limited to the study of biological systems either. Consider the PageRank algorithm which was first employed by Google (46). PageRank works by returning websites (represented as nodes) in order of their importance. Imagine, for website A its importance is a function of the number of websites linking to it—in this example two with B and C—and the importance of B and C determined by the number and importance of the websites linking to them. Using such a process enables all of the websites to be ranked relative to one another. Network propagation has the additional benefit of incorporating *a priori* information about the nodes, meaning that some nodes are more important than others and, as such, connections to these nodes are considered more favourable by the propagation algorithm, which can help to improve the identification of influential nodes by PageRank (47).

Here, we describe Esearch3D, an approach that exploits the global relationship between the 3D genome and transcription to identify active enhancers in 3C data by propagating gene expression levels across chromatin networks. Biologically, signals pertaining to transcriptional activation are transferred across the regulatory network from enhancers found in intergenic loci to genes in the form of transcription factors, cofactors and various types of transcriptional machinery such as RNAPII. Conceptually it can be described as a flow of information between enhancers and genes, mediated by the chromatin communication network. How and where this information is transmitted to and from is central to decoding the regulatory landscape of any gene and, therefore, identifying enhancers. We show that by reverse engineering this flow of information, we can identify intergenic regulatory enhancers using solely gene expression and 3D genomic data.

## MATERIALS AND METHODS

### From 3C to networks

Processed 3C datasets were downloaded for mouse embryonic stem cells (mESCs, serum) and three immune human cell types. For mESCs, we used the DNase I capture dataset from Joshi *et al.* (48) and promoter-capture Hi-C (PCHi-C) from Schoenfelder *et al.* (49) and reprocessed by Pancaldi *et al.* (32), both aligned to the GRCm38/mm9 reference genome. For the human primary immune cells, we used data from Javierre *et al.*, aligned to the GRCh37/hg19 reference genome (50). Contacts were normalized and loops were called in the three datasets using the CHiCAGO algorithm (51). The normalized contact matrix was then transformed into an unweighted and undirected network G = (V, E) where the chromatin fragments are represented as nodes V and their interactions as edges E.

### Gene annotation

Each node in the network represents a distinct genomic locus each containing biological features that infer their functional properties. To identify intergenic enhancers, each node was annotated with genes from Ensembl version 75 (52).

### Enhancer annotation

As is common in all enhancer prediction models, the identification of a suitable ‘truth set’ of validated enhancers is non-trivial. Currently gold standard approaches such as CRISPR (clustered regularly interspaced short palindromic repeats) perturbation are limited in both the scalability to assess putative enhancer sequences genome wide and the availability of these data for multiple cell types. As there is no genome-wide gold standard for enhancer identification in mESCs, we identified several enhancer-associated features that could be used to label the intergenic nodes as enhancers and non-enhancers.

We first used genome-wide chromatin profiles to identify putative enhancers in mESCs based on histone marks, including H3K27ac, H3K4me1 and H3K4me3 (Supplementary Table S1). To efficiently summarize the data, we utilized the popular ChromHMM method (53), and only bins with a posterior probability >0.95 were considered (54). The nodes were then labelled with the chromatin state bins and the percentage overlap of each state was calculated per fragment. This was further supplemented with the annotation of enhancer-associated features from experimental data; these included three RNAPII variants and P300 ChIPseq (54), STARR-seq (55) and bidirectional CAGE-seq sites from the FANTOM project (16,56). By combining these data, we were then able both to distinguish intergenic nodes as enhancers and to stratify this set into high and low confidence groups based on the number of features present. For the immune cells, we defined putative enhancers using chromatin states (Supplementary Table S2) from BLUEPRINT (57), eQTL SNPs (20) and bidirectional CAGE-seq sites from the FANTOM project (16,56).

### Gene expression data

Normalized gene expression single-cell RNAseq data for mESCs was downloaded from ESpresso (58) (https://espresso.teichlab.sanger.ac.uk), where we took the mean expression per gene of the cells in serum. The expression of CD4+ T cells, monocytes and neutrophils was obtained from the BLUEPRINT population dataset (20). The un-normalized read counts were re-normalized separately for each cell type using DESeq2 (59), keeping chromosome X (in the original publication, X and Y chromosomes were removed before normalization and the three cell types were normalized together). Finally, we took the mean gene expression for all the individuals.

### Development of a network-based expression propagation strategy

Our network-based propagation algorithm, implemented within Esearch3D, uses the propagation function *f*- defined by Zhou *et al.* (60). This algorithm is best understood as simulating a process where node A in the network contains an *a priori* value (Supplementary Figure S1). In the first iteration, the propagation algorithm spreads or ‘propagates’ this value to the adjacent neighbours of A, which are B, C and D. In the second iteration B, C and D can propagate their values from the first iteration to their adjacent neighbours E and F. This process continues for a set number of iterations based on the user input. These processes have a wide range of applications, but fundamentally they are all able to impute information in nodes with no *a priori* value based on the starting value at A.

Another feature of this algorithm is that the propagation processes factors in the global topology of the network. The number of connections between the nodes and their patterns will ultimately influence the final imputed value of each node. This can be useful when we want to assess the importance of the topological characteristics of nodes in the CIN in relation to gene expression. In our case, we wanted to understand if enhancers were uniquely connected within the network to transmit regulatory information to genes. To test this, we calculated an imputed activity score (IAS) in intergenic nodes based on the topology of a CIN using gene expression as our *a priori* starting values in gene nodes.

### Network-based propagation algorithm

We define a connected weighted graph G(V, E) with a set of nodes V = {v1, v2, …, vN} and a set of links E = {(vi, vj)|vi, vj∈V}, a set of source/seed nodes S⊆V and an N × N adjacency matrix W of link weights equal to 1 (Supplementary Figure S1). Here, we use a Random Walk with Restart (RWR) algorithm for measuring the relative importance of node vi to S. RWR mimics a walker that moves from a current node to a randomly selected adjacent node or goes back to source nodes with a back-probability γ∈(0, 1). RWR can be formally described as follows:


$$\begin{equation*}{\rm{Pt}} + {\rm{1}} = \left( {{\rm{1}} - {\rm{\gamma }}} \right){\rm{W^{\prime} Pt}} + {\rm{\gamma P0}}\end{equation*}$$

where Pt is an N × 1 probability vector of |V| nodes at a time step t of which the ith element represents the probability of the walker being at node vi∈V, P0 is the N × 1 initial probability vector and W' is the transition matrix of the graph; the (i,j) element in W' denotes a probability with which a walker at vi moves to vj among V{vi}.

To provide some intuition as to how this method works, consider the following example. At a genic node, a walker starts with some amount of information; in this case, the value given by the gene expression. The walker then has an equal probability to transition to any of its direct neighbours and carry a proportion of the gene expression value to that node. The amount of gene expression that can transition is determined by the insulating parameter γ. For example, where γ = 0.8, only 20% of the original gene expression value can transition. Low values of γ were chosen as genes are unlikely to be highly regulated by nodes very far away. The other parameter corresponds to the number of iterations and influences how many times this process takes place. A low number of iterations limits the amount of gene expression that can be carried to neighbouring nodes which can lead to these nodes obtaining similar values, making it more difficult to distinguish their relative importance to the gene node of interest.

### Esearch3D R package

Esearch3D is implemented in R, freely available at https://github.com/InfOmics/Esearch3D and https://doi.org/10.5281/zenodo.7737123. It takes as input a CIN and a cell type-specific gene expression profile. The network must be divided into two components, one composed of genes and the 3C fragments they map to where the overlap is represented as an interaction. The second component includes the interactions (contacts) from the 3C data. The network can be inputted as an adjacent matrix, edge list or igraph object (https://igraph.org). The gene expression profile must be in the standard matrix format N × 1 such that rows are genes and columns are the replicates, conditions. Esearch3D propagates the gene expression values into genic- and intergenic-fragments of the network in parallel using the R package doParallel (version 1.0.17). The functions ‘create_net2plot’ and ‘start_GUI’ represent results as an R igraph object and visualize it in a shiny (version 1.7.2) graphical user interface (GUI).

Esearch3D enables researchers to investigate genes or fragments by providing the names of the genes to analyse and running a new propagation with the function called ‘rwr_SGprop’ (Supplementary Figure S2). This step provides how much each gene of interest contributed to give information to the chromatin network nodes (genomic fragments) and also how much information each fragment received from the genes of interest. Esearch3D documentation includes a vignette detailing the workflow to classify any node as an enhancer or not, where information about the number of enhancer annotations associated with the fragments of the CIN are made available. This workflow enables the user to build a machine learning algorithm to classify nodes within the network as enhancers. Esearch3D computes the centrality measures of the nodes with the function ‘get_centr_info’, runs the multigene propagation and combines all the information in a matrix format. The subsequent matrix contains, for each node in the network, the IAS, the provided centrality measures as well as the number of enhancer annotations (provided by the user). The user can then build the classifier with the function ‘enhancer_classifier’. The function ‘train’ tunes and tests a Random Forest classifier with the R package mlr3. The best classifier can then be passed to the function ‘explain_classifier’ that builds an explainer with the R package DALEX. The explainer describes what the classifier learnt, how much each feature contributes to the prediction and which decisions are used to predict an unlabelled node. Topological features are calculated with the igraph R package and the Random Forest models are built with the DALEX R package (version 2.4.1)—see vignettes at https://github.com/InfOmics/Esearch3D for details.

### Precision–recall curves

Precision–recall curves plot, at increasing thresholds, the precision, which is the fraction of nodes correctly predicted as enhancers (i.e. positive for the five enhancer features in Figure 3A), and the recall, which is the fraction of the total number of enhancers predicted. Often, a trade-off occurs between these two metrics whereby increased recall reduces the precision, and vice versa. When plotted on the same graph, the area under the curve produced by this plot indicates the general classification performance of the model. The precision and recall of enhancer nodes using centrality measures and the corresponding area under the curve (AUPRC) were calculated using the precrec library (version 0.12.7) (61). The baseline values were calculated as the total number of enhancer nodes as a proportion of the total number of nodes in the CIN.

### Comparison of Esearch3d with the ABC model

The code to run ABC was downloaded from the GitHub repository (https://github.com/broadinstitute/ABC-Enhancer-Gene-Prediction). We used the DNaseI-seq and H3K27ac ChIPseq data from Joshi *et al.* (48), and the Hi-C data from Bonev *et al.* (62). DNaseI-seq and H3K27ac data required a few pre-processing steps to extract the input required for ABC. Reads were aligned to the reference mm9 genome file using bwa-mem (version 0.7.17). Picard (version 2.27.5) was used for sorting the aligned reads by coordinate and removing polymerase chain reaction (PCR) duplicates through the MarkDuplicates function. Bedtools (version 2.30.0) was used to retain only uniquely mapping reads using the -bq 30 parameter on samtools view. We ran the ABC model following the guidelines on the tool GitHub page from step one to three, changing the reference to mouse. In the makeCandidateRegions –nStrongestPeaks parameter, we used 150 000 as suggested by the authors. In the prediction step, we did not define a threshold (–threshold 0) to retrieve all the candidate regions with their respective scores. Precision–recall curves on ABC scores were computed in the same way as described in the previous section but using only three of the enhancer features, i.e. those that were not included as input of the model, namely enhancer features P300 ChIPseq (49), STARR-seq (50) and CAGE-seq (16,51). The same curves were computed on the Esearch3D IAS score and on the Esearch3D IAS score corrected with H3K27ac information. This correction was defined as follows: if the fragment node had <40% of its bases overlapping an acetylated region, then its IAS was set to 0.

## RESULTS

### Overview and conceptual description of Esearch3D

We have developed an R package called Esearch3D. The tool utilizes a bespoke semi-supervised network propagation algorithm to predict active enhancers using solely gene expression and chromatin interaction data. Enhancers are non-randomly located and connected in the network such that they have the potential to regulate cell type-specific gene expression. Esearch3D exploits this phenomenon to identify intergenic regions with regulatory functions.

Esearch3D models 3C data as CINs. Each genomic fragment from the 3C data is represented as a node and each fragment to fragment interaction as an edge (Figure 1). The fragment nodes are categorized into two types: gene-fragment nodes where the fragment coordinates overlap with a gene annotation and intergenic-fragment nodes where they do not. Additionally, Esearch3D includes a third node type: gene-expression nodes. Each gene-expression node is connected to the corresponding gene-fragment nodes by an edge where the gene-fragment node harbours a gene promoter. Given that genes can overlap with multiple fragments and multiple genes can overlap with the same fragment, integrating gene expression with fragment nodes is not a trivial step. Representing gene promoters as additional distinct nodes in the CIN enables Esearch3D to easily integrate gene expression profiles with minimal bias.

**Figure 2.**
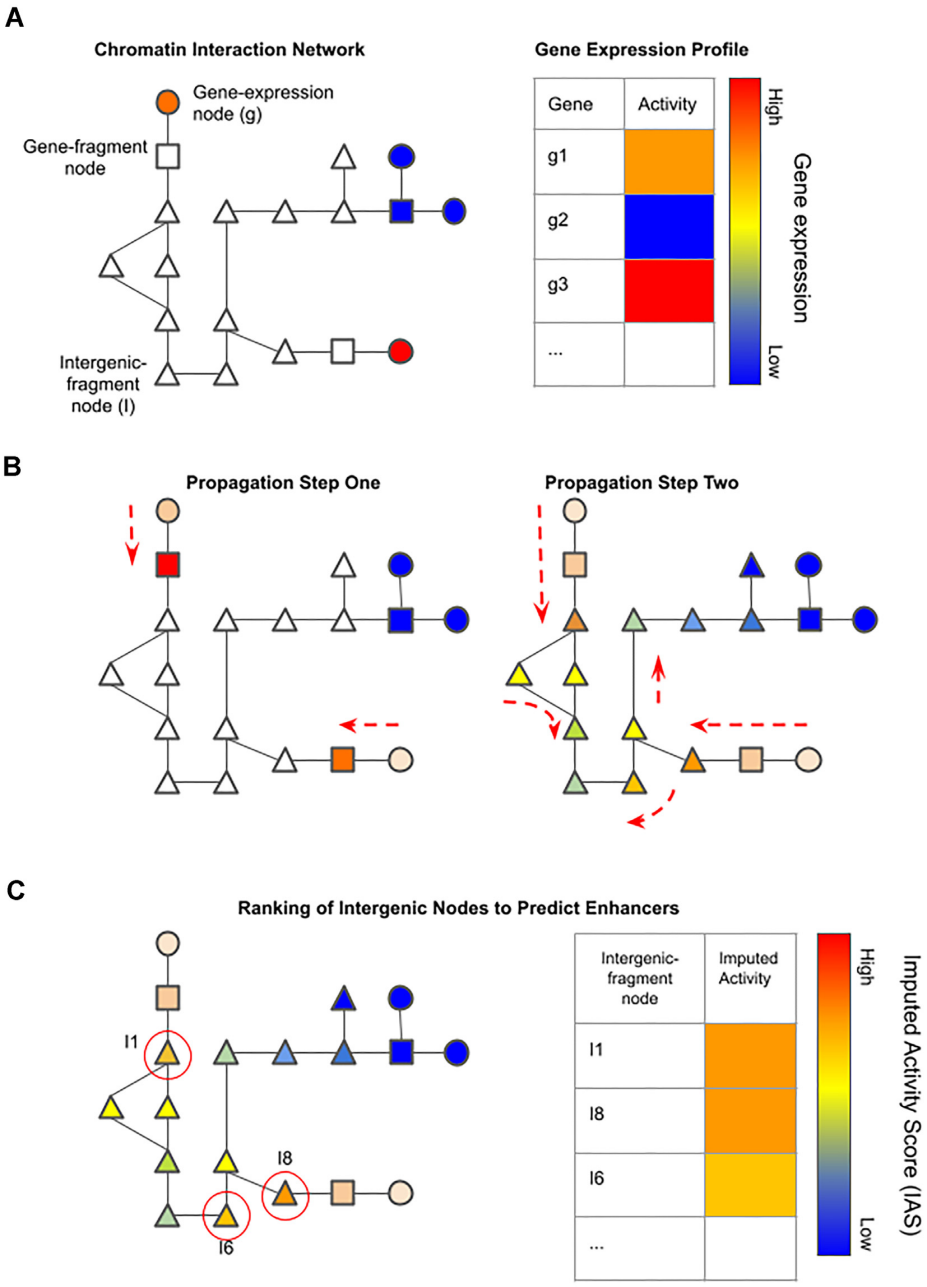
Schematic diagram of the network propagation used by Esearch3D to impute activity values at intergenic nodes. (**A**) Gene-expression nodes (circles) are mapped to gene-fragment nodes (square). Each gene-expression node (g) has an associated gene activity value determined by RNA-seq data. (**B**) Gene expression values are propagated from gene-expression nodes to gene-fragment chromatin nodes in propagation step one. Activity scores are then imputed in intergenic-fragment (I) chromatin nodes by propagating the scores from genic chromatin nodes. (**C**) Ranking of intergenic-fragment nodes by the imputed activity score to identify high confidence enhancer nodes.

Network-based propagation is a method used to predict the activity of nodes based on the activity of other nodes in the network. The basic idea is to use the known activity of each gene-fragment node and propagate this information throughout the network to all other nodes where proximal and tightly clustered nodes will receive a higher score than those further away in the network. Using this approach, Esearch3D maps the gene expression values to the corresponding gene-expression nodes and propagates them to their corresponding gene-fragment nodes in the first step, and to all other nodes, including intergenic-fragment nodes, in the second step (see example with a toy network in Figure 2). The resulting intergenic nodes receive an activity score (IAS, see Material and Methods) which accounts for the distance of the nodes, the topology of the network and the *a priori* expression values of all genes within the network (Figure 2C). For example, an intergenic-fragment node receives a high IAS, and is therefore more likely to be an enhancer if it is adequately connected and in close proximity to highly expressed gene-expression nodes. Much like gene expression reflects the transcriptional activity of a gene, the IAS reflects the enhancer activity of non-coding regions. The IAS score is based on the propagation of all expressed genes throughout the whole network. It is therefore a function of the distance of the intergenic-fragment nodes (i.e. the number of steps) to all other gene-fragment nodes in the network, the topology of the network and the expression levels of all genes within the network. In addition to predicting enhancers, our method highlights the integral role of global chromatin organization in mediating gene expression.

### Imputed activity scores at intergenic nodes are higher in active enhancer nodes

**Figure 3.**
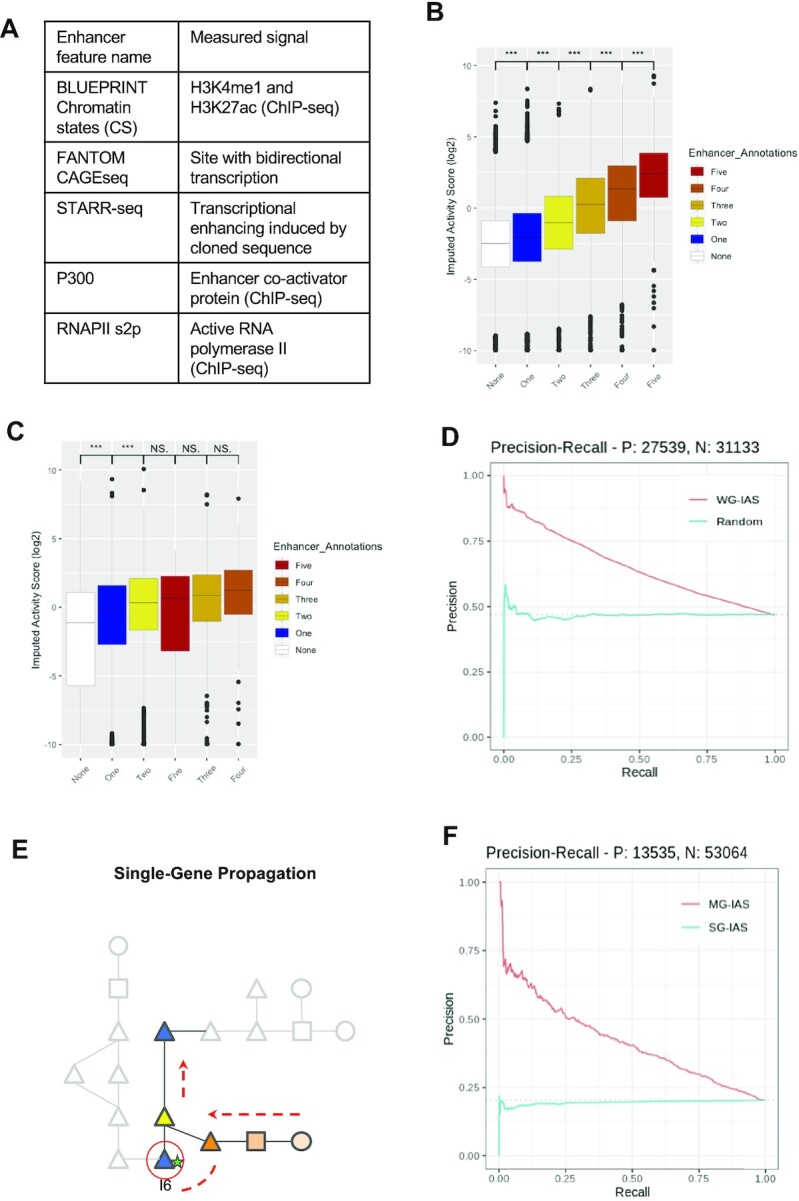
Enrichment of the Esearch3D IAS in enhancer annotated nodes. (**A**) Enhancer-associated features in mESCs (see the Materials and Methods for details). (**B**) Intergenic nodes in the mESC DHS-CHi-C network are stratified into enhancer and non-enhancer groups based on the number of enhancer-associated features that were annotated previously. The IAS is then plotted along the *x*-axis for each group of nodes. Boxplots are ordered by the median of each group and asterisks indicate significance in Wilcoxon tests (*P-value <0.05, **P-value <0.01 ***P-value <0.001). (**C**) The IAS in the mESC PCHi-C network by the number of enhancer annotations. (**D**) Precision–recall curves measuring the overall performance of the IAS in the prediction of enhancer nodes containing a minimum of one enhancer feature in the real (red line) and randomized (blue) networks. (**E**) A schematic of how single-gene propagation differs in the relative imputed activity scores. (**F**) Precision–recall curves for the multigene propagation (MG-IAS, red) versus the single gene propagation (SG-IAS, blue). For single gene propagation, we iteratively propagated the top 100 genes by expression and tested the predictive performance of MG-IAS on the intergenic nodes that receive a score. The multigene propagation performs with an AUPRC of 0.41, while the single gene propagation AUPRC is 0.19 over a baseline of 0.20.

Esearch3D infers the likelihood of enhancer activity at an intergenic locus with the IAS following the propagation of gene expression. Esearch3D requires two inputs: 3C data which are modelled as a network, and gene expression data. We generated our first set of results using the high-resolution DNase I Capture Hi-C (DHS-CHi-C) generated by Joshi and co-workers (48) in mESCs. This dataset was chosen owing to a diverse set of publicly available canonical enhancer-associated features in mESCs, which can be used to validate our predictions (Figure 3A). DHS-CHi-C targets regions of the mouse genome that are hypersensitive to DNase I (DHS regions), enriching for chromatin-accessible regions. From this DHS-CHi-C data, we created a CIN, where each genomic fragment from this dataset is represented as a node and each fragment–fragment interaction as an edge (Figure 1). The DHS-CHi-C network consists of a total of 162 615 fragment nodes connected by 787 879 edges with an average degree of 9.69. The CIN is split across 776 connected components. The majority of the nodes (97.7%) are members of the major connected component. We then identified gene-fragment nodes by mapping Ensembl-defined genes to the nodes (see the Materials and Methods and Supplementary Tables S3–S5). We found that 36% of the nodes in the CIN were labelled as intergenic; that is to say that there are no overlaps of the gene body [including untranslated regions (UTRs), exons and introns]. Although introns also have many enhancers [see (63), for example], we focused here in the prediction of enhancers not overlapping with genes.

To validate the efficacy of the IAS as a predictive feature of intergenic enhancers, intergenic-fragment nodes were then labelled using the following enhancer-associated features: chromatin states derived from histone mark ChIP-seq experiments, P300, RNAPII s2p elongating variant (54), STARR-seq (55) and CAGE-seq data from the FANTOM project (16,56) (see the Materials and Methods and Figure 3A). In the mESC DHS-CHi-C, network we found that in nodes that contain at least one enhancer-associated feature there was a significant difference in the median value of the IAS in enhancer intergenic-fragment nodes compared with non-enhancer intergenic-fragment nodes (Wilcoxon test *P*-value <2.2e-16). Intergenic-fragment nodes were then stratified into six groups based on the different combinations of the five enhancer labels as well as the absence of any labels. We found that the median value of the IAS in each group increased as the number of enhancer labels increased, while the median IAS from nodes devoid of any enhancer annotations to the group labelled with all five annotations increased by a factor of 46 (Figure 3B). These results show that the value of the IAS is higher in intergenic-fragment enhancer nodes compared with non-enhancer nodes. Furthermore, the IAS is found to be higher in nodes that contain an increasing number of enhancer features (Figure 3B).

We then applied Esearch3D to an alternative 3D genome capture data type by extending the analysis to PCHi-C-derived interactions for mESCs. The resulting network showed that PCHi-C results in a higher number of connected components resulting in a more disconnected network than the DHS-CHi-C network (Supplementary Tables S3 and S4). Despite this, results are consistent with our previous observations using the DHS-CHi-C network with a higher IAS in enhancer nodes and significant increases in the median value of IAS between each group (Wilcoxon rank sum test *P*-value <2.2e-16, Figure 3C). The IAS shows a minimum 14-fold enrichment overall in the high confidence enhancer nodes compared with the non-enhancer nodes for the mESC PCHi-C network.

These results show that Esearch3D can be used to approximate enhancer activity in chromatin fragments derived from DHS-CHi-C and PCHi-C experiments. This is achieved independently from any *a priori* knowledge of enhancer-associated features, using only gene expression and 3C contact data and without the need for model parameterization. These results show that Esearch3D can be used to leverage the intrinsic relationship between the chromatin topology and gene expression to identify which nodes harbour enhancers.

### Imputed activity scores can be used to classify enhancer nodes

Following the observations of IAS enrichment in enhancer nodes, we then used the IAS to classify nodes as enhancers and non-enhancers. Using the canonical enhancer-associated features (Figure 3A) as our ground truth, we plotted the precision–recall curves for the DHS-CHi-C network and determined the area under the precision–recall curve (AUPRC, Materials and Methods). The IAS is able to correctly classify an enhancer node containing at least one of the five enhancer-associated features with an AUPRC of 0.650 over a baseline of 0.469 (Figure 3D). The baseline is calculated as the ratio of enhancer nodes (*n* = 27 539) divided by the total number of intergenic nodes (*n* = 58 672). This is equal to a performance of 0.181 over baseline. For the mESC PCHi-C network there is a 0.095 increase over the baseline (Supplementary Figure S3), a 0.086 decrease when compared with the mESC DHS-CHi-C network. The differences observed between the two capture methods are likely to be due to the smaller representation of the mESC genome by PCHi-C compared with a DHS-CHi-C that covers 50% more of the total genome. This has two main effects. One is the number of potential enhancers that are captured; the proportion of intergenic nodes in the DHS-CHi-C network labelled as enhancers is 50% compared with 43% for the PCHi-C network. The second is that a lower coverage capture of the genome by the PCHi-C results in the capture of fewer enhancers across the genome. In the PCHi-C network, we observe a lower average degree of 2.61 compared with 9.69 for the DNase I network (Supplementary Table S4). We note that this does not drastically alter the density of the network which calculates the ratio between the number of actual connections versus the number of potential connections; the number of potential connections is given by the binomial coefficient of the number of nodes. This suggests that the PCHi-C is not losing information about the topology of the network, rather it is sampling a smaller proportion of the real CIN.

### Chromatin topology and gene expression are crucial features to identify enhancers

The ability of Esearch3D to predict enhancer nodes is based on the propagation of gene expression across the CIN. This propagated gene expression data from the genic nodes to the intergenic nodes. Our results suggest that enhancer nodes maintain distinct connectivity patterns with gene nodes compared with non-enhancer nodes such that they receive a higher IAS. Therefore, the quality of the predictions relies on both the gene expression data and the network topology.

To demonstrate this, we tested how the performance of the IAS as a predictive feature changes when the topology of the network is perturbed. To do so, we applied a degree-preserving rewiring algorithm to shuffle the edges of the mESC DNase I network to create an ensemble of 100 rewired networks. This maintains the number of connections a node has while shuffling the node to which it is connected. We then ran Esearch3D on the ensemble of randomized networks to predict enhancer nodes. We then aggregated and compared the predictions with those of the original network. Results showed that following the randomization the ability of the model to classify enhancer nodes was lost with an AUPRC = 0.49 over a baseline of 0.47 (Figure 3D). This indicates that the spatial proximity of intergenic nodes with genic nodes is important for driving these predictions, in line with evidence suggesting the role of transcriptional factories is a key determinant of gene expression (64).

There is increasing evidence that gene expression is regulated at a global level as outlined by the pan-genomic and omnigenic models (38,40). These models suggest that aggregate and subtle changes in the expression of genes can influence the expression of others and, by extension, enhancers can have subtle cascading effects on transcription throughout the genome. For Esearch3D we modelled this process by propagating the expression of all genes across the network and calculating the IAS at each intergenic node, which reflects its regulatory contribution and therefore its likelihood of containing an enhancer. To provide a counterfactual example, we propagated the expression of a single gene of interest in order to predict the enhancers that directly regulate its expression. This approach is also available in the Esearch3D software. In this scenario, the expression profile of a single gene of interest was propagated across the CIN. We carried out this test on the top 100 most highly expressed genes (112 when including ties). Results showed that the propagation of 112 single genes across the network reduced the performance of the model compared with that of a random classifier AUPRC = 0.19 (Figure 3F). Together, these results indicate that the global properties of the chromatin network and the global effects of gene expression are important features to identify enhancers.

In addition we also investigated how Esearch3D compares with the ABC model (25). ABC scores enhancer–gene interactions based on enhancer activity, chromatin accessibility, gene expression and, optionally, 3D interactions. Both ABC and Esearch3D use gene expression and 3D genome data to predict enhancers. However, ABC requires additional input information in the form of ChIPseq data for H3K37ac, ATACseq or DNaseI-seq and, optionally, full-genome Hi-C data. In order to compare the two tools, we took into account their dissimilarities in terms of required input data. We compared the precision and recall of the ABC scores and Esearch3D’s IAS in mESCs, using three enhancer features for validation that neither of the two methods use as input, namely P300 ChIPseq (49), STARR-seq (50) and CAGE-seq (16,51).

We show how Esearch3D and ABC have very comparable results in terms of AUPRC, with 0.412 for ABC and 0.397 for Esearch3D. Importantly, ABC uses two data types that Esearch3D does not, DNaseI-seq and H3K27ac, to pre-define the regions of highest potential of being active enhancers. This means that using a restricted number of input resources, Esearch3D is still able to perform comparatively. Running the ABC model is less convenient in terms of required input data, and at the same time more cumbersome, as it requires further data pre-processing. These results suggest that using further input data in Esearch3D can improve its performance. Indeed, when we pre-select nodes which present acetylated regions in Esearch3D, we also obtain better precision–recall curves than ABC, with an AUPRC of 0.445 (Supplementary Figure S4).

### Esearch3D can be used to study the regulatory function of regions with eQTL SNPs

**Figure 4.**
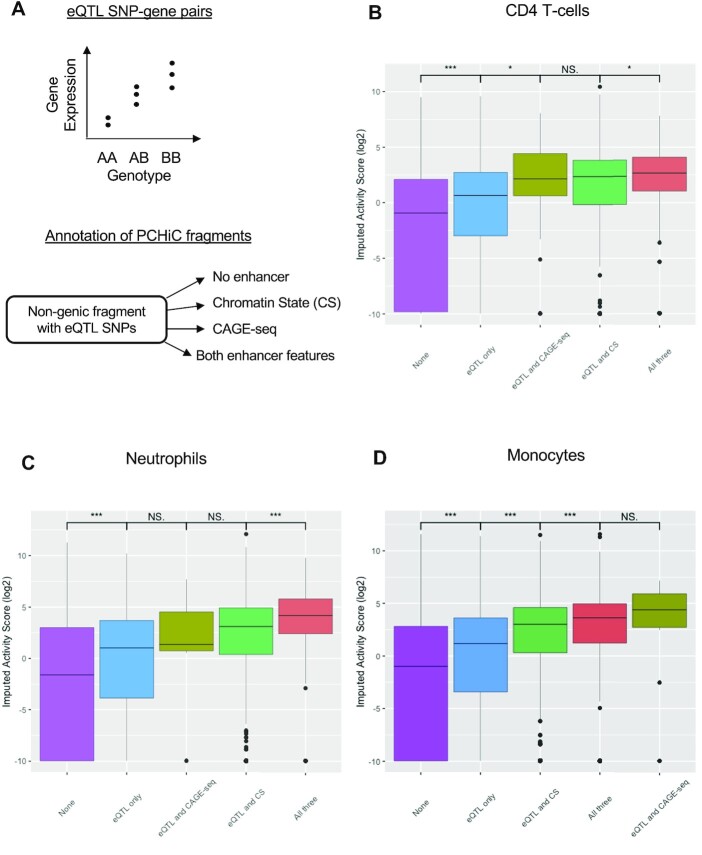
Enrichment of the Esearch3D IAS in non-genic regions with eQTL SNPs. (**A**) Schematic of how eQTL SNPs are detected and annotation of nodes in the networks of human immune cells. Boxplots are ordered by the median of each group and asterisks indicate significance in Wilcoxon tests (*P-value <0.05, **P-value <0.01 ***P-value <0.001). (B–D) IASs in (**B**) CD4+ T cells (**C**) neutrophils and (**D**) monocytes.

One of the main lessons gained from GWAS is that most disease-associated SNPs lie within non-coding regions that are enriched in enhancers (65). eQTL studies have shown how genetic variation across the genome can result in significant and quantifiable changes in gene expression (66). eQTL SNPs are defined by classifying individuals by that SNP genotype and calculating correlations with gene expression levels of genes in proximity to their linear genome (Figure 4A). These approaches help to identify the cell types where disease-associated SNPs show an effect and to statistically associate non-coding SNPs with the potential gene targets, thereby aiding in the functional interpretation of GWAS hits outside genes (21).

The BLUEPRINT Consortium generated eQTL data for three human immune cell types: monocytes, neutrophils and CD4+ T cells (20). We used these data to test the ability of Esearch3D to prioritize the regulatory potential of non-genic regions harbouring eQTL SNPs. For these cell types, we used available PCHi-C data (50) to generate 3D genome networks and annotated the non-genic nodes with the cell type-specific eQTL SNPs, enhancer chromatin states from BLUEPRINT (57) and bidirectional CAGE-seq sites from the FANTOM project (16,56). We found 46 078 monocyte nodes, 39 930 neutrophil nodes and 48 016 T-cell nodes with eQTL SNPs, but only 41% of eQTLs in monocytes overlapped with nodes containing either FANTOM or BLUEPRINT enhancers, with 28% in neutrophils and 31% in T-cells.

Interestingly, we observed a significantly higher IAS in non-genic nodes with eQTLs compared with non-genic nodes without eQTLs in all three cell types (*P*-value <2.2e-16), even in nodes that did not overlap with experimentally defined enhancer features (Figure 4B–D). These eQTLs may point to loci that contain weaker enhancers that are not annotated by either CAGE-seq or chromatin state data. However, Esearch3D also predicts higher activity (IAS) when the non-genic nodes with eQTL SNPs also overlap with enhancer features (*P*-value <2.2e-16) (Figure 4B–D). These results suggest that the IAS calculated by Esearch3D can help to prioritize eQTL SNPs with higher potential of being regulatory regions.

In line with previous results in mESC-derived networks, we also find that the IAS is generally higher in nodes with all three annotations. This further highlights the generalizability of our model to additional cell types, including primary cell types that are difficult to assay for enhancer annotations that depend on transfections, such as CRISPR deletion/silencing experiments or STARR-seq screens.

### The IAS can be used in conjunction with centrality measures to classify enhancer nodes

Employing five commonly used network measures (67), we calculated different topological characteristics of intergenic nodes in the mESC DHS-CHi-C network (Supplementary Table S5). We calculated the degree, which reflects the number of edges, or connections, that the nodes in the network maintain; the betweenness centrality, which measures the number of shortest paths that pass through a node—this measurement reflects how important a node is in connecting other nodes; the closeness centrality that reflects how central a node is in the network relative to all other nodes; the clustering coefficient, a measure of the degree to which nodes in a graph tend to cluster together; and finally the eigenvector centrality, which measures the influence of a node within the network—this is calculated relative to the scores of other nodes in the network where a node with a high eigenvalue score connects to many other nodes with high eigenvalue scores.

**Figure 5.**
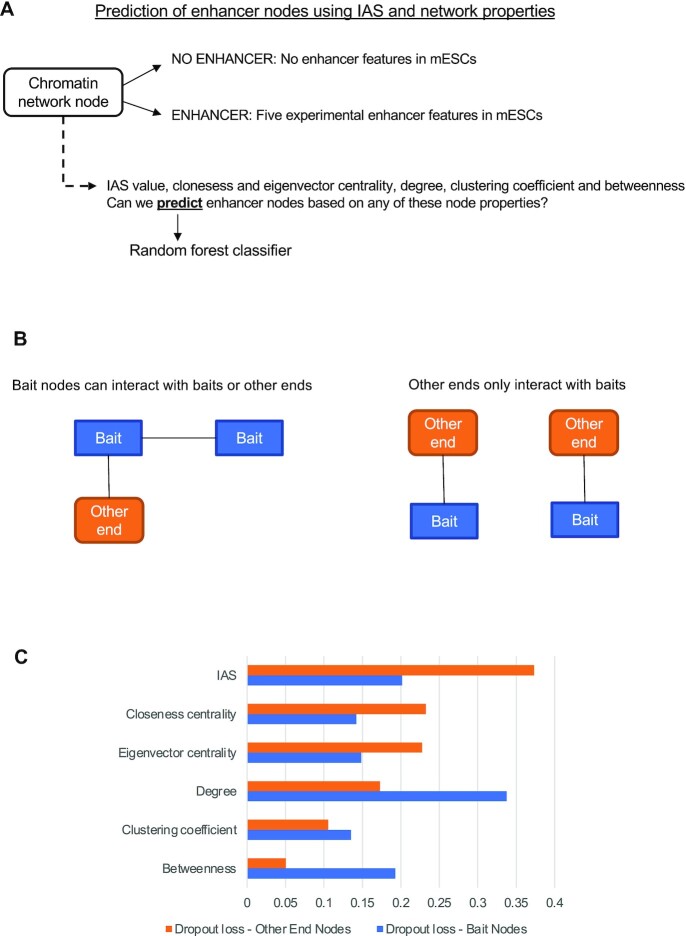
The Esearch3D IAS and other network metrics can be used to predict enhancers. (**A**) Approach to classify enhancer and non-enhancer nodes. (**B**) Cartoon illustrating the different connectivity of bait (blue) and other end (orange) nodes in capture Hi-C, resulting in different network metrics. (**C**) The dropout loss of topological network measures used to build a Random Forest classifier of enhancer nodes (predicting the presence of at least one enhancer feature) with baits (blue) and other end (orange) nodes of the DHS-CHi-C network.

A Random Forest classifier was then used to combine these scores along with the IAS in order to classify intergenic enhancer nodes from the mESC DHS-CHi-C network. We utilized the out-of-bag (OOB) error as a measurement to evaluate the contribution of each of the five topological metrics along with the IAS when classifying intergenic enhancer and intergenic non-enhancer nodes. OOB measures the loss of classification performance of the Random Forest classifier when the feature of interest is removed. For capture Hi-C there is a bias between baits (such as promoters or, in this case, DHS sites) and other ends (non-bait fragments) due to the enrichment protocol (Figure 5B). This results in artificially lower connectivity for other end nodes when compared with baits (32). Due to the unique nature of capture Hi-C networks, we evaluated the five topological metrics and the IAS for non-genic baits and other ends separately, using two different Random Forest models.

We found that for non-genic baits, the degree centrality was the feature that was the most informative when classifying nodes into intergenic enhancers and intergenic non-enhancers, with a drop out loss of 0.33 (Figure 5C). The IAS was the next best measure, with a drop out loss of 0.20. For other ends, the best performing feature was the IAS, with a drop out loss of 0.37 (Figure 5C). Interestingly, the degree centrality was only the fourth most important feature for the other ends where connectivity information is lost due to the capture Hi-C design. In summary, the IAS performs well for both types of fragments and contributes to enhancer prediction, highlighting the advantage of integrating gene expression data compared with only measuring the topological characteristics of intergenic nodes. This approach is particularly powerful to predict enhancers in less connected other end nodes, suggesting that the Esearch3D approach is a useful tool to compensate for the interaction sampling bias in capture Hi-C experiments.

### Esearch3D is implemented as an R package with a shiny-based GUI to explore the results

We have shown how the IAS can be used to predict the location of enhancers. We have implemented Esearch3D as an R package. The method can be applied following the provided workflow with the CIN and the gene expression profiles of the user's cell type of interest (Supplementary Figure S2). The software begins by performing the function ‘rwr_OVprop’ twice. During the first step, the expression of the genes is propagated from their corresponding nodes into the genic nodes. In the second step, the gene expression is then propagated across the rest of the network. This imputes a proxy for transcriptional activity, the IAS, at intergenic nodes.

**Figure 6.**
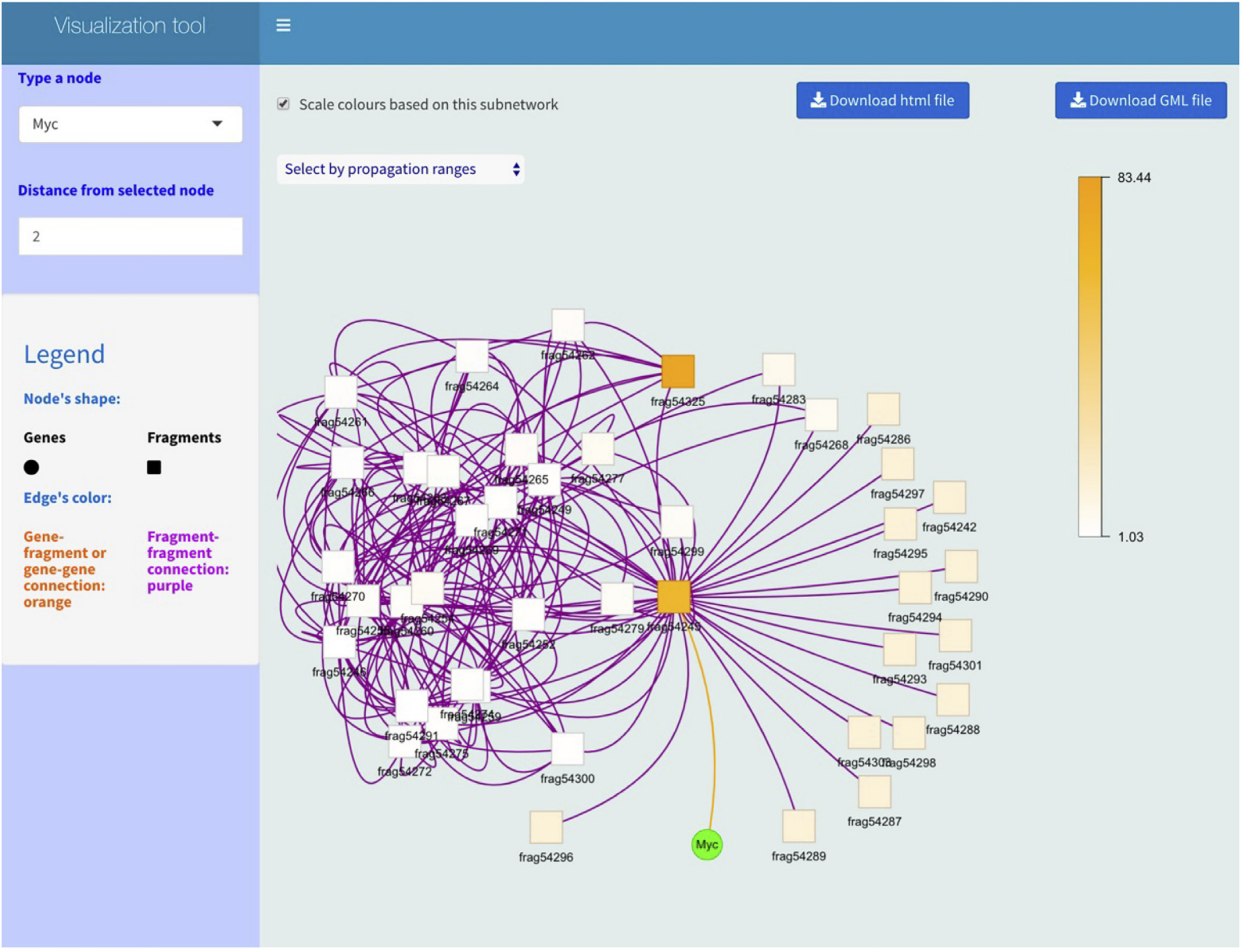
Visual exploration of Esearch3D results. The R shiny app provides a GUI to explore subnetworks with the multigene propagation results, which can be exported in Cytoscape format. The screenshot shows the direct interaction of the *Myc* gene (represented as a node in the network, as explained in Figure 1) with the DHS-CHi-C fragment containing it and direct interactors of that fragment (plus their interactions). The white to orange key corresponds to the Esearch3D IAS value of the fragments (scaled based on the visualized subnetwork).

The user can visualize the propagation results in a matrix format or with the GUI developed with R shiny (Figure 6). The GUI allows one to explore a gene expression profile after a network-based propagation, to investigate the IASs obtained by specific genes and their neighbourhoods and to import the propagated chromatin network into Cytoscape (68). The GUI allows users to visualize and focus on specific modules of the CIN based on their interests. To illustrate the GUI functionality, we loaded the propagation results of the mESC DHS-CHi-C network and explored the interactors of the gene node of the proto-oncogene *Myc* (Figure 6). The interaction (in orange) between the *Myc* node and node frag54325 indicates that the *Myc* locus is located inside this genomic fragment in mouse chromosome 15; the purple edges show the direct fragment–fragment interactions of the fragment containing *Myc*. The interacting fragment with the highest IAS value is frag54325 (chr15:61807659–61821282): this corresponds to a genomic region downstream of *Myc* that overlaps with all enhancer features (STARR-seq, FANTOM bidirectional CAGE-seq, P300, enhancer chromatin states and RNAPII s2p). Interestingly, this genomic region overlaps with the GSDMC gene cluster that is downstream of the recently discovered ′blood enhancer cluster’, conserved between human and mouse, that controls *Myc* in mouse haematopoietic stem cells and progenitors (69). The region containing the GSDMC gene cluster has been shown to form part of the regulatory network of *MYC* in human cell lines (70), suggesting that this could be an important regulatory node in mESCs too.

## DISCUSSION

The folding of chromatin into hierarchical structures ultimately acts to localize genes with the prerequisite machinery for transcription such as RNAPII (32,71) and the appropriate chromatin modifications (72). Whether gene expression dictates the chromatin architecture or vice versa is poorly understood. In either case, it is clear that the organization of chromatin plays an important role in mediating the communication between genes and enhancers. Herein, we have outlined a method that can be used to predict enhancers by leveraging the relationship between gene expression and the global chromatin architecture. To achieve this, we model interaction data from Hi-C-derived methods as networks.

We use a network propagation algorithm based on random walks to integrate gene expression data within the CIN. The use of network propagation has been proposed previously in other fields, such as heat diffusion processes, energy states within electrical circuits, and graph kernels in machine learning (42). Other applications in biology include gene function prediction, module discovery and drug target prediction; see Cowen *et al.* (42). In cancer research, for example, network propagation has been used to identify previously unknown proteins involved in the progression of cancers by propagating mutational frequencies across a protein–protein interaction network (73). We use the same concept with chromatin–chromatin interaction networks and propagating gene expression values instead of mutational frequencies. By doing so, Esearch3D is able to impute an activity score (IAS) in intergenic nodes by propagating the gene expression values from genic nodes across the CIN. The IAS is a metric that summarizes the importance of an intergenic node in the context of its proximity to all of the genic nodes within the network, the relative expression of those genes as well as the topology of the entire network. Most widely used methods to identify enhancers do not incorporate gene expression, nor do they evaluate the subtle, but important, influences exerted by chromatin's global organization. Esearch3D builds on this, modelling 3D genome data as networks, and then exploits graph-theory algorithms to integrate RNA-seq data in order to calculate an IAS.

The underlying assumption of Esearch3D is that the activity, defined by the gene expression, of one chromatin fragment is influenced by, or can influence, the activity of all other chromatin fragments. Transcriptional factories occur through dynamic processes that lead to phase separation (64). These are, in part, influenced by relative levels of gene expression across the CIN, suggesting global influence at a local level (74). We too identify such behaviour in our model. We propagate the expression of all of the genes simultaneously in order to model the global interdependent relationships between genes, chromatin conformation and enhancers. Indeed, we find that the performance of Esearch3D is driven by propagating the expression of all genes, whereas a poor performance is seen when propagating the expression of a single gene. This would suggest that gene expression and chromatin organization are linked at a global level.

The challenges that exist in finding enhancers stem from a fundamental gap in our knowledge about their composition and mode of action. It is becoming increasingly apparent that chromatin architecture plays an important role in gene regulation by localizing enhancers to gene promoters in specific patterns. This idea is commensurate with the looping mechanisms that localize enhancers and gene promoters resulting in an increased contact frequency (3). These effects can be obvious as shown by the loss of interaction between the ZRS enhancer and the *SHH* promoter following the deletion of a key CTCF site (75). This suggests that enhancers and genes are distinctly connected within the 3D chromatin structure compared with other loci. However, to what extent 3D interactions are always essential for gene regulation is debatable. Some studies have shown that the disruption of some TADs does not lead to wide-scale changes to gene expression (76), suggesting that mechanisms other than the chromatin topology may regulate gene expression. Alternatively, complex networks are known to be robust to attack (77) and, as such, deletion of interactions within the network should not always be expected to result in large gene expression changes. Other explanations such as redundancy in the enhancer networks are equally plausible (6,7). In such cases, more care should be taken when making claims about the role of chromatin organization in gene regulation when regions are studied in isolation. In fact, we show that by studying the relationships between gene expression and the global structure of chromatin, we can accurately identify enhancer regions. We have shown that enhancers and genes within our networks tend to contain distinct topological features, as shown by the centrality scores when compared with non-enhancer and intergenic nodes, and that these features can be used to predict intergenic enhancer nodes. Our data would therefore suggest that chromatin organization plays an important role in coordinating gene regulation.

## DATA AVAILABILITY

Esearch3D is implemented in R, freely available at https://github.com/InfOmics/Esearch3D and https://doi.org/10.5281/zenodo.7737123.

## Supplementary Material

gkad229_Supplemental_File
